# Supplementary material for: Modeling the effects of Aedes aegypti’s larval environment on adult body mass at emergence
Source: PLoS Comput Biol. 2021 Nov 22;17(11):e1009102. doi: 10.1371/journal.pcbi.1009102 (PMC8608295; doi:10.1371/journal.pcbi.1009102)
Supplement: S1 File — A mathematical description of the full system, along with exemplary equations are provided. In Table A, for each fitted parameter, we list the fitted value under the variation considered. A dash indicates that parameter was not fitted under that variation. (PDF) [file pcbi.1009102.s001.pdf]

Modeling the effects of *Aedes aegypti*'s larval environment on adult body mass at emergence

Melody Walker[1,2], Karthikeyan Chandrasegaran[3], Clément Vinauger[3,4], Michael A Robert[5], Lauren M Childs[1,4,*]

1 Department of Mathematics, Virginia Tech, Blacksburg, Virginia, USA
2 Current address: Laboratory for Systems Medicine, University of Florida Health, Gainesville, Florida, USA
3 Department of Biochemistry, Virginia Tech, Blacksburg, Virginia, USA
4 Center for Emerging Zoonotic and Arthropod-Borne Pathogens, Virginia Tech, Blacksburg, Virginia, USA
5 Department of Mathematics and Applied Mathematics, Virginia Commonwealth University, Richmond, Virginia, USA

S1 File: Detailed model description, equations and Table A of fitted model parameters.

We developed a discrete time model (time step = 1 day) of an *Aedes aegypti* mosquito population which incorporates each biological stage of the mosquito's life cycle: four larval instars (LX, where X denotes the instar: 1, 2, 3, or 4), pupa (P), and adult (M and F). We divided each stages further by mass, which we denote using a subscript: small (s), medium (m), and large (l).

All mosquitoes begin in the $L1$ stage to mimic the initial condition of our experiments. $\beta(t)$ represents the number of $L1$ larvae of a given sex inputted in the system at time t. As we only have larvae enter the system initially, $\beta(t) = 0$ when $t \neq 0$. For model simulations of low density treatment, $\beta(0) = 26/2$, and of high density treatment, $\beta(0) = 78/2$. As we are unable to sex the individuals until emergence as adults, we assume that, in each replicate, 50% of the initial larvae are male, and 50% are female. Individuals develop through successive larval stages, where the proportion of juvenile mosquitoes that develops each day is governed by the function $f(N)$. See details on $f(N)$ in section 2.2.2. At each transition, a proportion of individuals die. See details on mortality $\mu(N)$ in section 2.2.3. As our model uses a discrete time step, we must select the order at which to apply events (transition and mortality) at the end of each period. Here, we allow individuals to transition first and then introduce mortality.

During the larval stages, mosquitoes may remain in their current mass group or transition to a higher mass group with the assumption that mosquitoes changing mass groups can only transition to the next highest mass group in a single time step. For example, during the transition from the first larval stage ($L1$) to the second larval ($L2$) stage, individuals can either stay in the small mass group or move to the medium mass group. Similarly, each individual in $L2$ that transitions to the third larval stage ($L3$) can stay in the same mass group or move to a higher mass group.

We do not track adult populations, but only record the emergence of adults by sex and mass group through time. For example, $F_s(t)$ represents the number of newly emerged females in the small mass group at time t, and $M_l(t)$ represents the number of newly emerged males in the large mass group at time t. Note that for the larval and pupal stages, we track males and females separately to account for differences in

development and survival that are sex-dependent, but we only present larval and pupal equations for a single sex here as the equations are structurally identical. While the equation structure is identical, the following functions differ depending on sex: death proportion ($\mu(N)$), the proportion which develop ($f(N)$), and the proportion which grow ($G_1(r/N), G_2(r/N)$), where N is total larvae (males + females) and r is resources.

Equations for the aquatic phases (identical for both male and female) are given by:

$$
\begin{aligned}
L1(t+1) &= \beta(t) + \big(1-\mu(N)\big)\big(1-f(N)\big)L1(t),\\
L2_s(t+1) &= \big(1-\mu(N)\big)\Big(\big(1-G_1(r/N)\big)f(N)L1(t) + \big(1-f(N)\big)L2_s(t)\Big),\\
L2_m(t+1) &= \big(1-\mu(N)\big)\Big(G_1(r/N)f(N)L1(t) + \big(1-f(N)\big)L2_m(t)\Big),\\
L3_s(t+1) &= \big(1-\mu(N)\big)\Big(\big(1-G_1(r/N)\big)f(N)L2_s(t) + \big(1-f(N)\big)L3_s(t)\Big),\\
L3_m(t+1) &= \big(1-\mu(N)\big)\Big(G_1(r/N)f(N)L2_s(t) + (1-G_2(r/N))f(N)L2_m(t)\\
&\qquad + \big(1-f(N)\big)L3_m(t)\Big),\\
L3_l(t+1) &= \big(1-\mu(N)\big)\Big(G_2(r/N)f(N)L2_m(t) + \big(1-f(N)\big)L3_l(t)\Big),\\
L4_s(t+1) &= \big(1-\mu(N)\big)\Big(\big(1-G_1(r/N)\big)f(N)L3_s(t) + \big(1-f(N)\big)L4_s(t)\Big),\\
L4_m(t+1) &= \big(1-\mu(N)\big)\Big(G_1(r/N)f(N)L3_s(t) + \big(1-G_2(r/N)\big)f(N)L3_m(t)\\
&\qquad + \big(1-f(N)\big)L4_m(t)\Big),\\
L4_l(t+1) &= \big(1-\mu(N)\big)\Big(G_2(r/N)f(N)L3_m(t) + \big(1-f(N)\big)L4_l(t)\Big),\\
P_s(t+1) &= \big(1-\mu(N)\big)\Big(\big(1-G_1(r/N)\big)f(N)L4_s(t) + \big(1-f(N)\big)P_s(t)\Big),\\
P_m(t+1) &= \big(1-\mu(N)\big)\Big(G_1(r/N)f(N)L4_s(t) + \big(1-G_2(r/N)\big)f(N)L4_m(t)\\
&\qquad + \big(1-f(N)\big)P_m(t)\Big),\\
P_l(t+1) &= \big(1-\mu(N)\big)\Big(G_2(r/N)f(N)L4_m(t) + \big(1-f(N)\big)P_l(t)\Big),
\end{aligned}
$$

and for emerging male adults:

$$
\begin{aligned}
M_s(t+1) &= \big(1-\mu(N)\big)f(N)P_s(t),\\
M_m(t+1) &= \big(1-\mu(N)\big)f(N)P_m(t),\\
M_l(t+1) &= \big(1-\mu(N)\big)f(N)P_l(t),
\end{aligned}
$$

and for emerging female adults:

$$
\begin{aligned}
F_s(t+1) &= \big(1-\mu(N)\big)f(N)P_s(t),\\
F_m(t+1) &= \big(1-\mu(N)\big)f(N)P_m(t),\\
F_l(t+1) &= \big(1-\mu(N)\big)f(N)P_l(t).
\end{aligned}
$$

In our model, we denote the second compartment in each of L4 and pupal stages with an asterisk. For example, P_l is the first compartment for pupae in the large mass group

and P_l^* is the second compartment. No growth is assumed during these transitions so they take the form

$$P_l^*(t+1) = \big(1 - \mu(N)\big) f(N) P_l(t).$$

Variation	k_m	k_f	μ^*	μ_f	μ_m	f	g_1	g_2	n_1	n_2
C1	0.7022	0.6442	0.1060	-	-	-	-	-	-	-
C2	0.8233	0.7346	0.0550	-	-	-	-	-	-	-
C3	0.9515	0.8491	0.0490	-	-	-	-	-	-	-
D1	0.9515	0.8491	0.0419	-	-	-	-	-	-	-
D2	0.9515	0.8491	-	0.0454	0.0380	-	-	-	-	-
D3	0.9515	0.8491	-	0.0661	0.0381	23.4982	-	-	-	-
E	0.9515	0.8491	-	0.0661	0.0381	23.4982	1.0000	0.5024	3.0970	8.0961

Table A. Fitted model parameters. For each fitted parameter, we list the fitted value under the variation considered. A dash indicates that parameter was not fitted under that variation.
