# Supplementary figures and images for: Modeling the effects of Aedes aegypti’s larval environment on adult body mass at emergence
Source: PLoS Comput Biol. 2021 Nov 22;17(11):e1009102. doi: 10.1371/journal.pcbi.1009102 (PMC8608295; doi:10.1371/journal.pcbi.1009102)

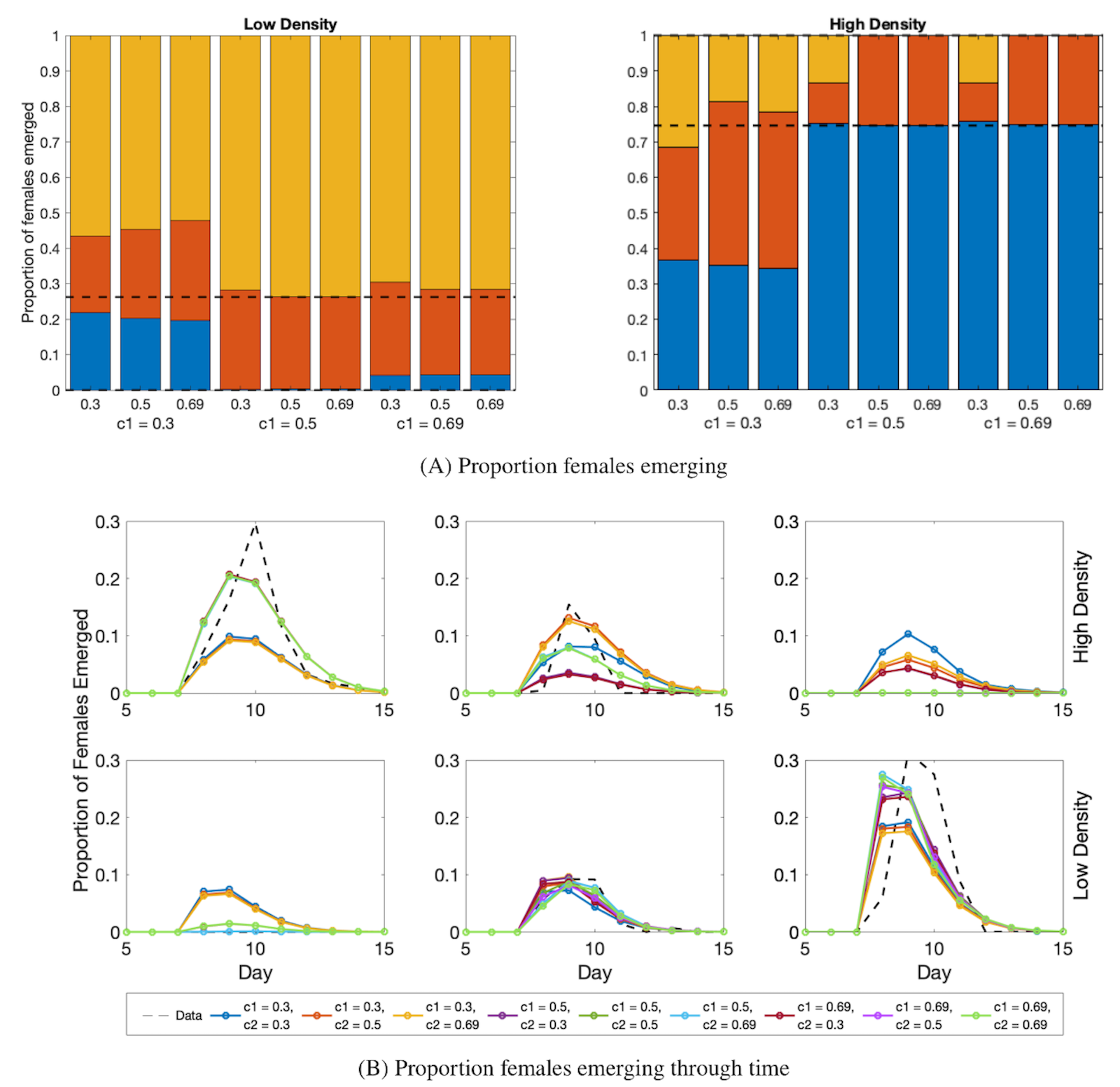

Supplement: S1 Fig — (A) The proportion of emerging females in each mass group as c1 and c2 vary for low density (left) and high density (right) treatments. Blue, red, and gold represent small, medium, and large mass groups, respectively. The black dashed lines indicate the divisions at which different mass groups were expected based on means of proportions of the mass groups from the data. In particular, the lower dashed line separates small and medium mosquitoes, and the upper dashed line separates medium and large mosquitoes. For close fits to the data, the blue bar would be below the lower dashed line, the red bar would be entirely between the two dashed lines, and the gold bar would be above the higher dashed line. (B) The proportion of females emerging over time by mass group: small (left), medium (middle), and large (right). The top row is the high density treatment, and the bottom row is the low density treatment. The solid color lines are model output with different c1 and c2 values. The black dashed line represents the mean of the data. (TIFF) [file pcbi.1009102.s002.tiff]

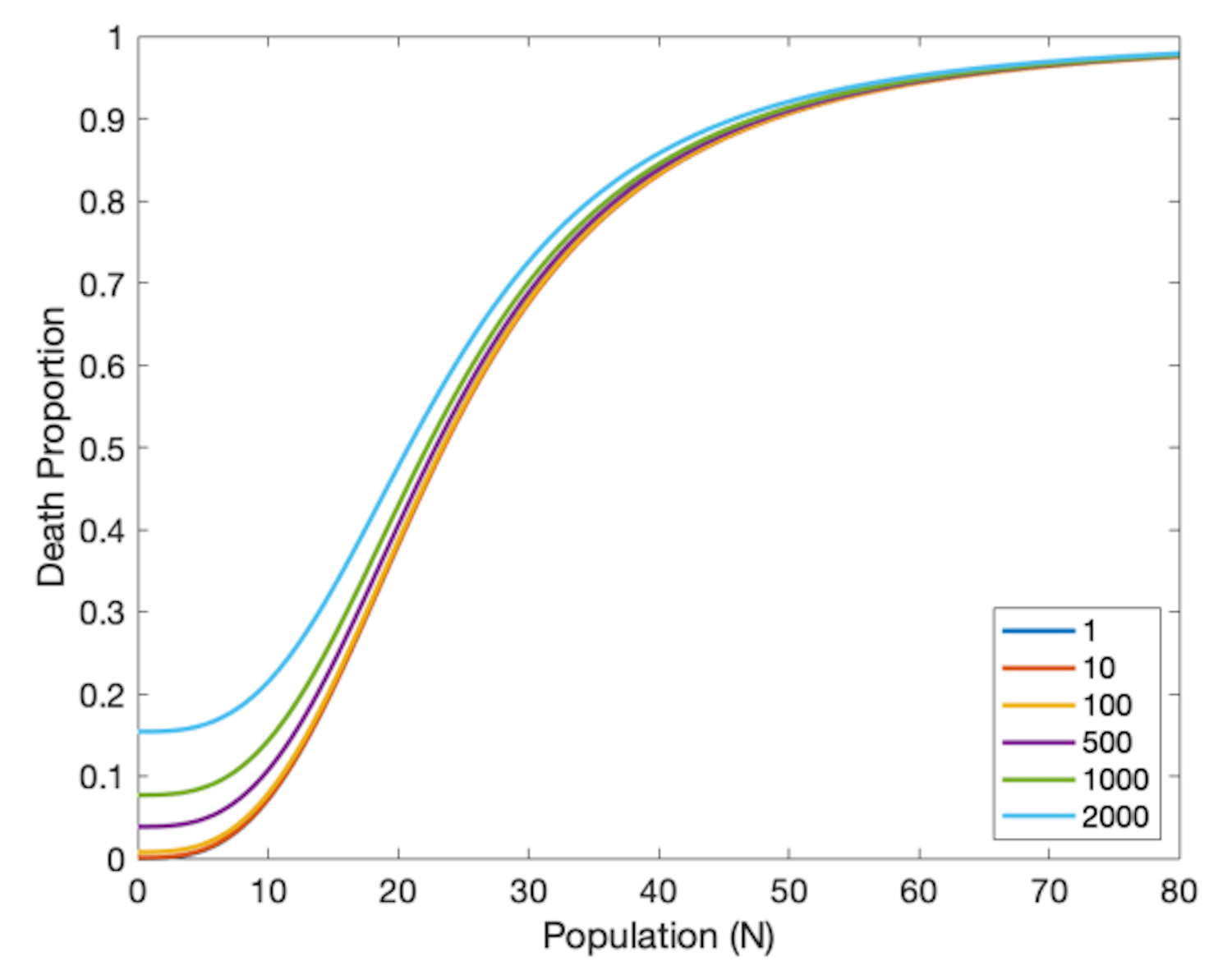

Supplement: S2 Fig — Density-dependent death function f(N)=μfN3+hN3+f3 with f = 23.5 and μf = 0.0661. The minimum constant h varies from 1 to 2000. See section 2.2.3 for details on the functional form. (TIFF) [file pcbi.1009102.s003.tiff]

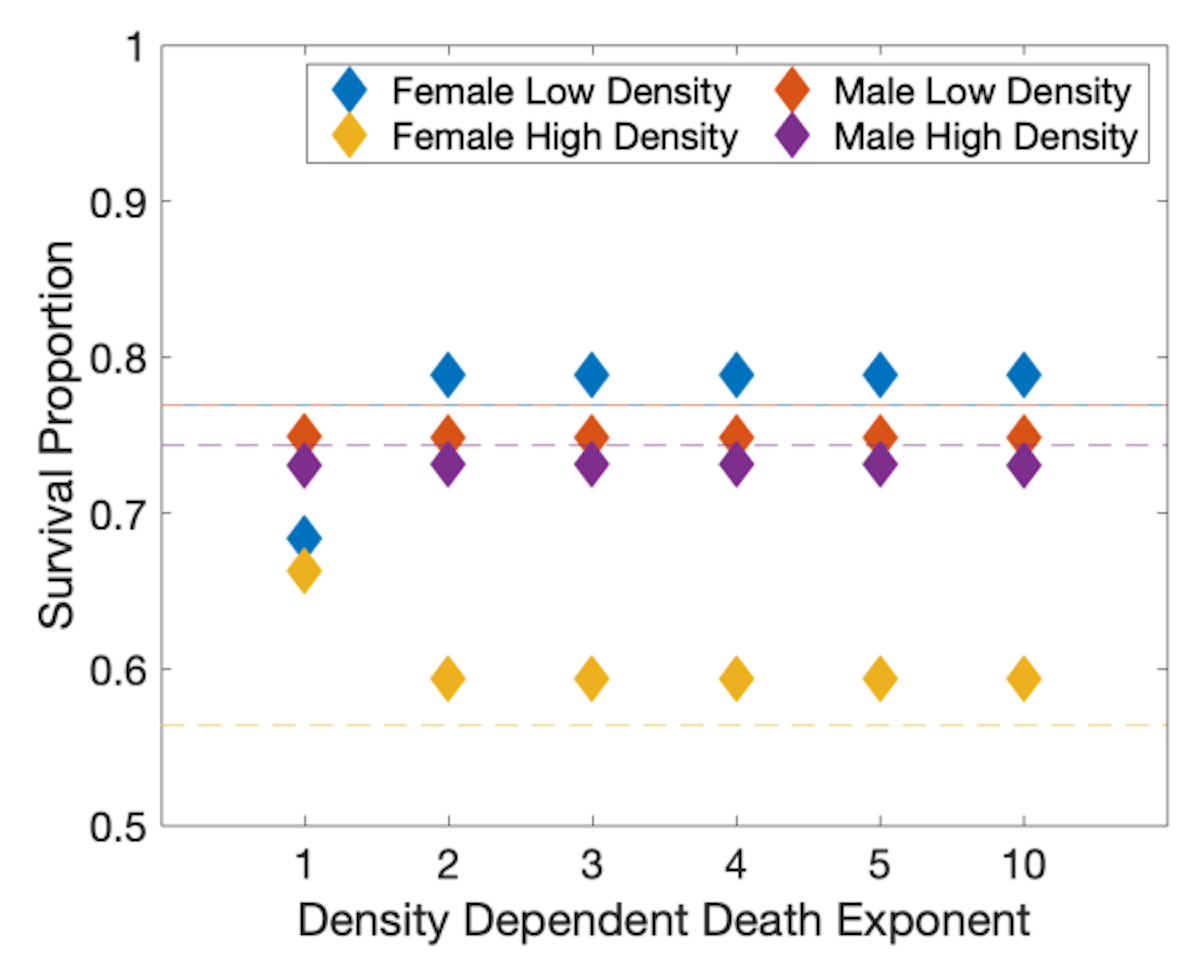

Supplement: S3 Fig — Model results employing the density-dependent death function f(N)=μfNn+hNn+fn with f = 23.5, μf = 0.0661, and h = 100. The density-dependent death exponent, n, varies along the x-axis from 1 to 10. The total larvae at a given time, N, changes in the course of the model simulations. The dashed lines represent the median values from the data and the diamonds the model results for the survival proportion of females in low density (blue), females in high density (yellow), males in low density (red), and males in high density (purple). The survival proportion for males and females in low density is indistinguishable in the data. See section 2.2.3 for details on the functional form of f(N). (TIFF) [file pcbi.1009102.s004.tiff]

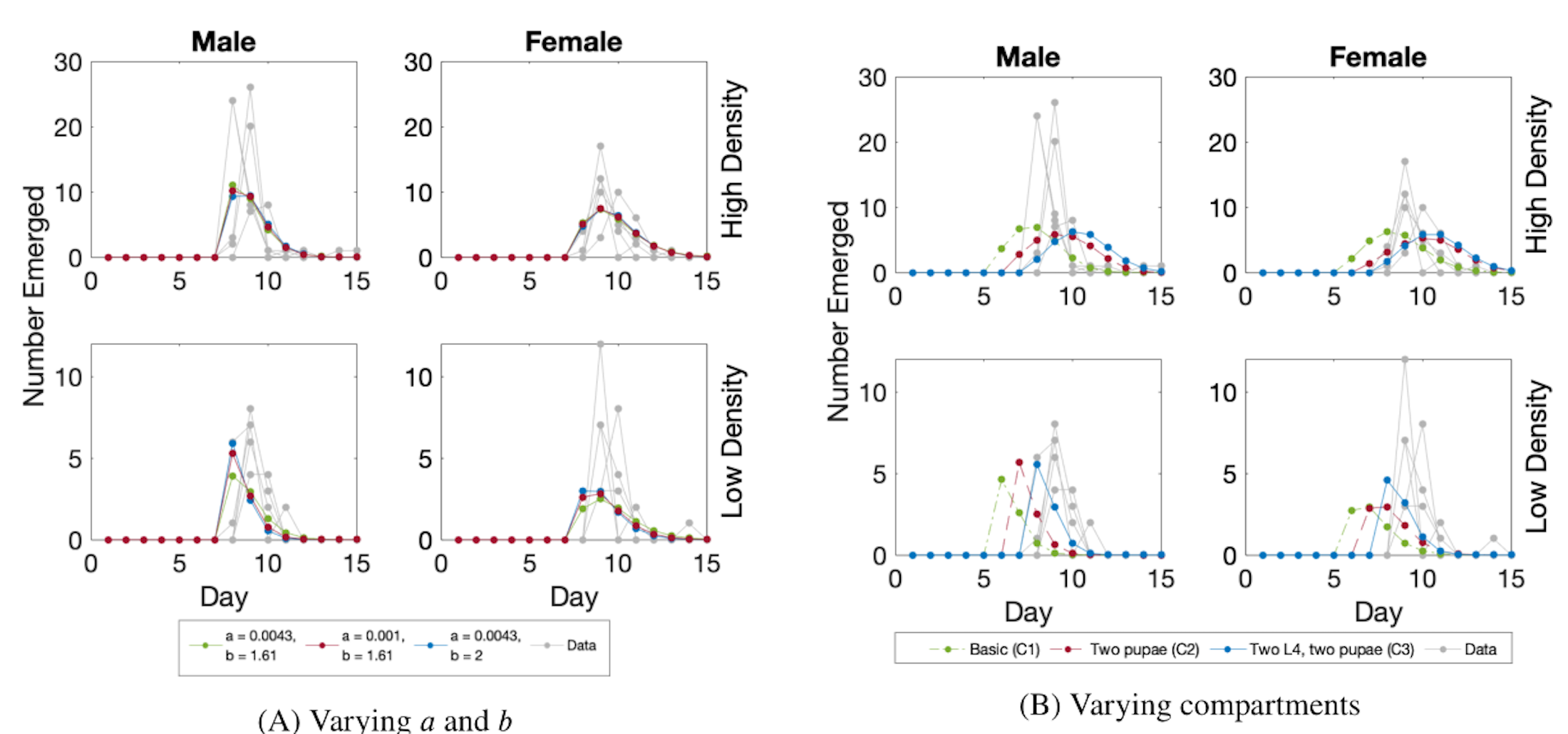

Supplement: S4 Fig — (A) The solid blue line shows the choice of our model with our original parameters a = 0.0043 and b = 1.61. The two other parameter choices with similar AICc values are shown, when a = 0.001 and b = 1.61 (solid green line) and when a = 0.0043 and b = 2 (solid dark maroon line). (B) This shows all three variations C1 (dashed dotted green line), C2 (dashed dotted maroon line), and C3 (solid blue line) with the parameters set at a = 0.01 and b = 1.61. (TIFF) [file pcbi.1009102.s005.tiff]
